# Pulmonary Sarcomatoid (Pleomorphic) Carcinoma Presenting As Secondary Spontaneous Pneumothorax in Diffuse Cystic Lung Disease: A Case Report

**DOI:** 10.7759/cureus.101633

**Published:** 2026-01-15

**Authors:** George K Annan, Abigail Mills-Annoh, Jeffrey Afrifa-Yamoah, Praise Ekakitie, Patrick O Berchie

**Affiliations:** 1 Internal Medicine, Piedmont Athens Regional Medical Center, Athens, USA; 2 Internal Medicine, Trinity Health Ann Arbor, Ann Arbor, USA; 3 Department of Pathology and Laboratory Medicine, Emory University School of Medicine, Atlanta, USA

**Keywords:** atezolizumab, cavitary lung lesion, diffuse cystic lung disease, immune-checkpoint inhibitors, non-small cell lung cancer (nsclc), pd-l1 expression, pleomorphic carcinoma of the lung, pulmonary sarcomatoid carcinoma (psc), robotic lobectomy, secondary spontaneous pneumothorax

## Abstract

Pulmonary sarcomatoid carcinoma is a rare and aggressive subtype of non-small cell lung cancer associated with poor prognosis and atypical clinical presentations. Spontaneous pneumothorax as an initial manifestation is exceedingly uncommon and may obscure the underlying diagnosis, particularly in patients with diffuse cystic lung disease.

This report describes a case of pulmonary sarcomatoid (pleomorphic) carcinoma presenting with secondary spontaneous pneumothorax in the setting of diffuse cystic lung abnormalities, highlighting the diagnostic challenges encountered and the importance of maintaining suspicion for malignancy in atypical presentations. Early tissue diagnosis, multidisciplinary evaluation, and molecular profiling played key roles in guiding definitive management.

This case underscores the need to consider lung cancer in patients presenting with spontaneous pneumothorax and atypical parenchymal findings, as timely recognition may permit curative-intent treatment and biomarker-informed systemic therapy in this aggressive malignancy.

## Introduction

Pulmonary sarcomatoid carcinoma (PSC) represents a rare and highly aggressive subset of non-small cell lung cancer (NSCLC), accounting for less than 1% of all lung malignancies [1,2]. These tumors are defined by the presence of poorly differentiated epithelial carcinoma with sarcomatoid features, including spindle and/or giant tumor cells. Among the recognized histologic variants, pleomorphic carcinoma is the most common and consists of conventional NSCLC components admixed with at least 10% spindle or giant cells [1-3].

PSC is strongly associated with tobacco exposure and is characterized by rapid growth, early invasion, high metastatic potential, and poor overall prognosis. Most patients present with advanced or metastatic disease at the time of diagnosis [4-6]. Unusual clinical presentations can further delay recognition and treatment. Spontaneous pneumothorax as an initial manifestation of lung cancer is rare, and when present, it is often associated with advanced disease or poor outcomes [7,8].

Diffuse cystic lung disease adds additional diagnostic complexity, as it broadens the differential diagnosis to include smoking-related emphysema, pulmonary Langerhans cell histiocytosis, lymphangioleiomyomatosis, and autoimmune-associated interstitial lung diseases [9-11]. In this report, we describe a case of pleomorphic-type PSC arising in a diffusely cystic lung and presenting initially as a secondary spontaneous pneumothorax, highlighting diagnostic challenges and management considerations.

## Case presentation

A 56-year-old woman with no established chronic medical conditions presented to the emergency department with a three-day history of progressive dyspnea and mild right-sided chest discomfort. She denied fever, cough, hemoptysis, weight loss, or night sweats. She reported approximately 20 years of daily cigar smoking, averaging two "black and mild" cigars per day, corresponding to a heavy cigar-smoking exposure. She had not received regular medical care for several decades. Alcohol use was occasional and social, without binge or heavy drinking patterns. There was no known family history of lung disease or malignancy.

On presentation, she was hemodynamically stable and breathing comfortably on room air. Physical examination revealed markedly diminished breath sounds over the right hemithorax without tracheal deviation. The remainder of the examination was unremarkable. Laboratory studies, including complete blood count and basic metabolic panel, were within normal limits.

**Figure 1:**
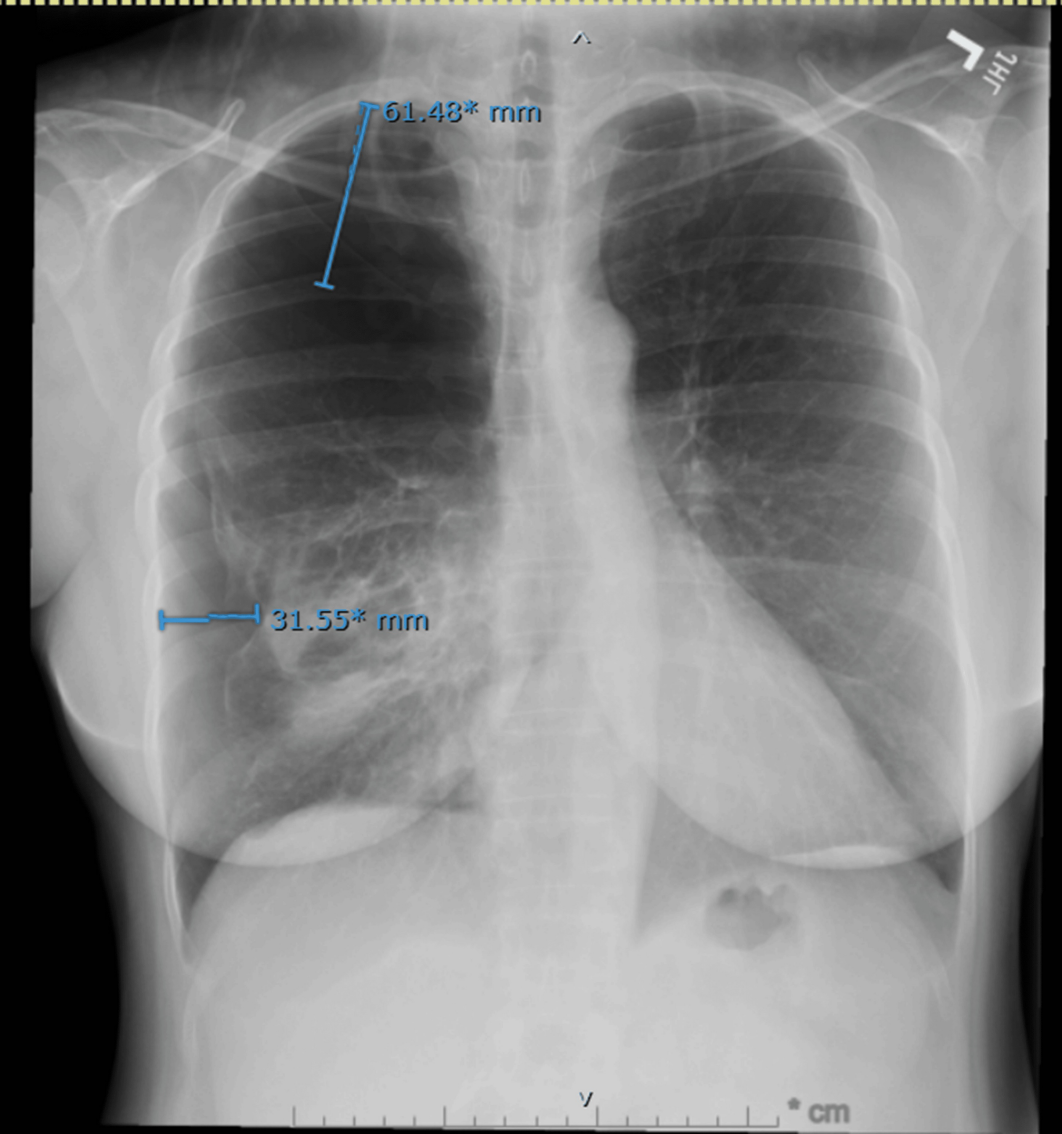
Initial chest radiograph demonstrating a large right-sided spontaneous pneumothorax Posteroanterior chest radiograph showing a large right-sided spontaneous pneumothorax with pleural separation measuring up to approximately 6 cm superiorly and 3 cm laterally. There is no evidence of mediastinal shift. The left lung is clear, and the cardiac silhouette is normal in size.

**Figure 2:**
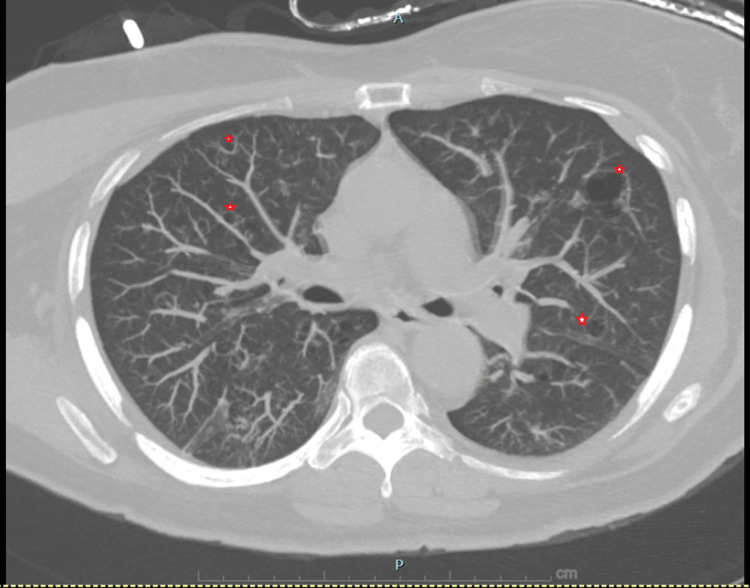
Chest CT demonstrating diffuse cystic lung disease Noncontrast chest computed tomography (lung windows) demonstrating multiple variably sized, thin-walled pulmonary cysts distributed throughout both lungs (red stars). This appearance is atypical for centrilobular emphysema and broadened the differential diagnoses to include cystic lung diseases such as pulmonary Langerhans cell histiocytosis, lymphangioleiomyomatosis, and autoimmune-related interstitial lung disease.

**Figure 3:**
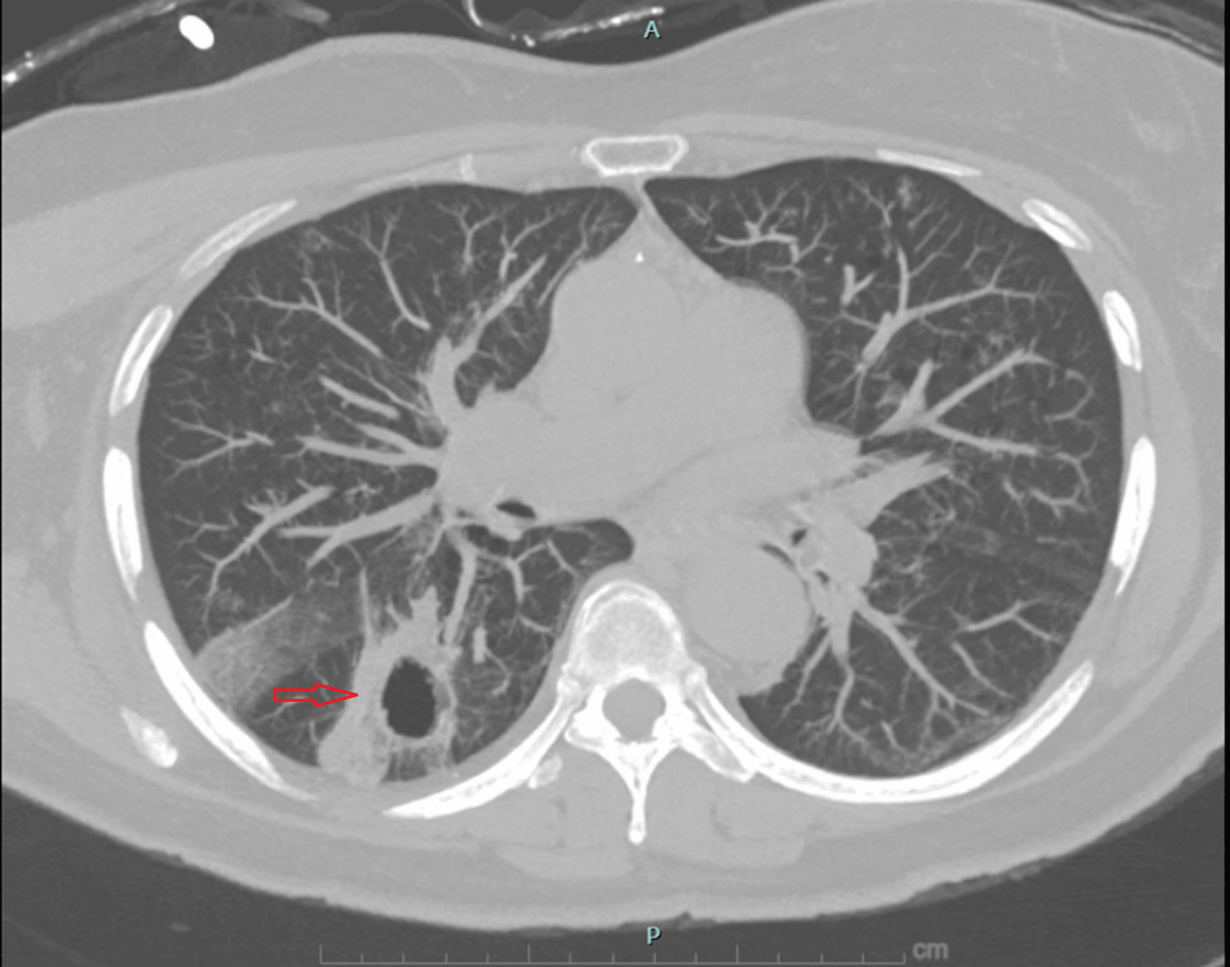
Thick-walled cavitary lesion in the right lower lobe on chest CT Noncontrast chest computed tomography (lung windows) demonstrating an irregular, thick-walled cavitary lesion in the right lower lobe measuring approximately 3 cm (red arrow). The imaging appearance raised concern for a neoplastic process versus infectious or inflammatory etiology and prompted further diagnostic evaluation.

Chest radiography demonstrated a large right-sided pneumothorax without mediastinal shift (Figure 1). A small-bore pleural catheter was placed, resulting in successful re-expansion of the lung. Non-contrast chest CT obtained during the admission revealed diffuse scattered thin-walled cysts throughout both lungs, an appearance atypical for centrilobular emphysema, as well as an irregular thick-walled cavitary lesion measuring approximately 3 cm in the right lower lobe (Figures 2,3). The differential diagnosis included infectious, inflammatory, autoimmune, and neoplastic processes.

Autoimmune serologies revealed a positive antinuclear antibody (1:80), elevated anti-Sjögren's Syndrome A (SSA) antibodies, and mildly positive anti-Sjögren's Syndrome B (SSB) antibodies, raising initial concern for an autoimmune or paraneoplastic process. However, the patient denied sicca symptoms, inflammatory arthritis, rash, or glandular enlargement. Complement levels were normal, antineutrophil cytoplasmic antibodies were negative, and rheumatologic evaluation did not support a clinically significant connective tissue or paraneoplastic autoimmune disease.

The patient underwent computer-assisted navigational bronchoscopic biopsy targeting the cavitary lesion. Cytology demonstrated a malignant spindle-cell neoplasm consistent with sarcomatoid carcinoma. Bronchoalveolar lavage was negative for bacterial, mycobacterial, and fungal pathogens. Immunostaining and serum testing for pulmonary Langerhans cell histiocytosis and lymphangioleiomyomatosis were negative.

**Figure 4:**
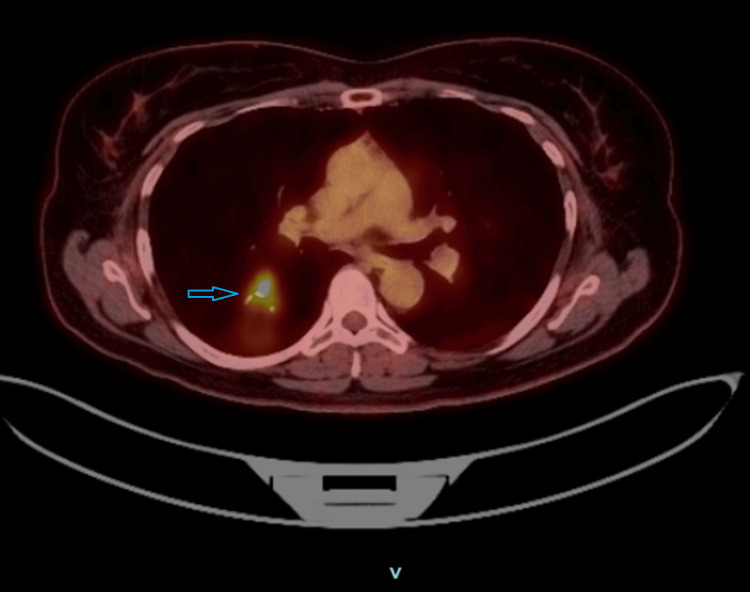
FDG PET/CT demonstrating a metabolically active cavitary right lower lobe lung mass 18F-fluorodeoxyglucose positron emission tomography/computed tomography (FDG PET/CT) demonstrating a cavitary mass (blue arrow) in the superior segment of the right lower lobe with nodular thickened walls and increased metabolic activity (SUVmax 5.6). No abnormal FDG uptake is identified in mediastinal lymph nodes.

Staging PET imaging demonstrated a hypermetabolic cavitary mass in the superior segment of the right lower lobe with a maximum standardized uptake value (SUVmax) of 5.6, without evidence of mediastinal or distant metastatic disease (Figure 4). Pulmonary function testing showed preserved spirometry with normal forced expiratory volume in one second and forced vital capacity, no obstructive pattern, and mildly reduced diffusing capacity (DLCO 66%), indicating adequate pulmonary reserve for surgical intervention.

**Figure 5:**
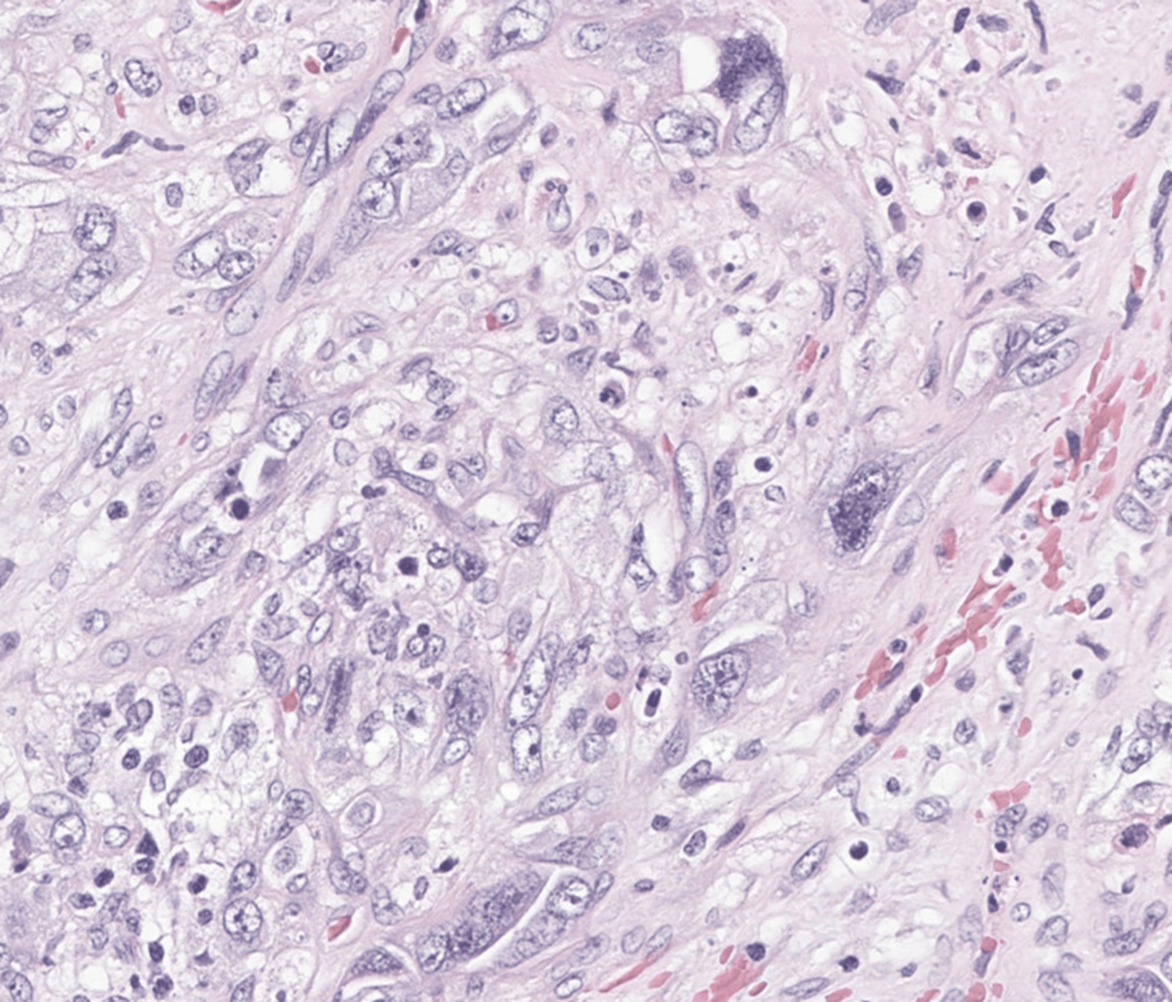
Pleomorphic giant tumor cells on histopathology Hematoxylin and eosin–stained section demonstrating pleomorphic giant tumor cells, characteristic of pulmonary sarcomatoid carcinoma.

**Figure 6:**
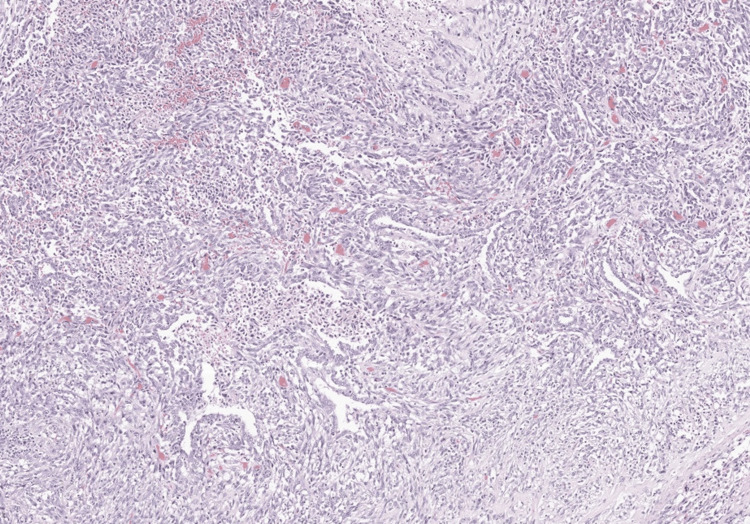
Admixed epithelial and spindle-cell components in pleomorphic carcinoma Hematoxylin and eosin–stained section showing malignant epithelial elements admixed with spindle-cell components, a defining histologic feature of pleomorphic (sarcomatoid) carcinoma.

**Figure 7:**
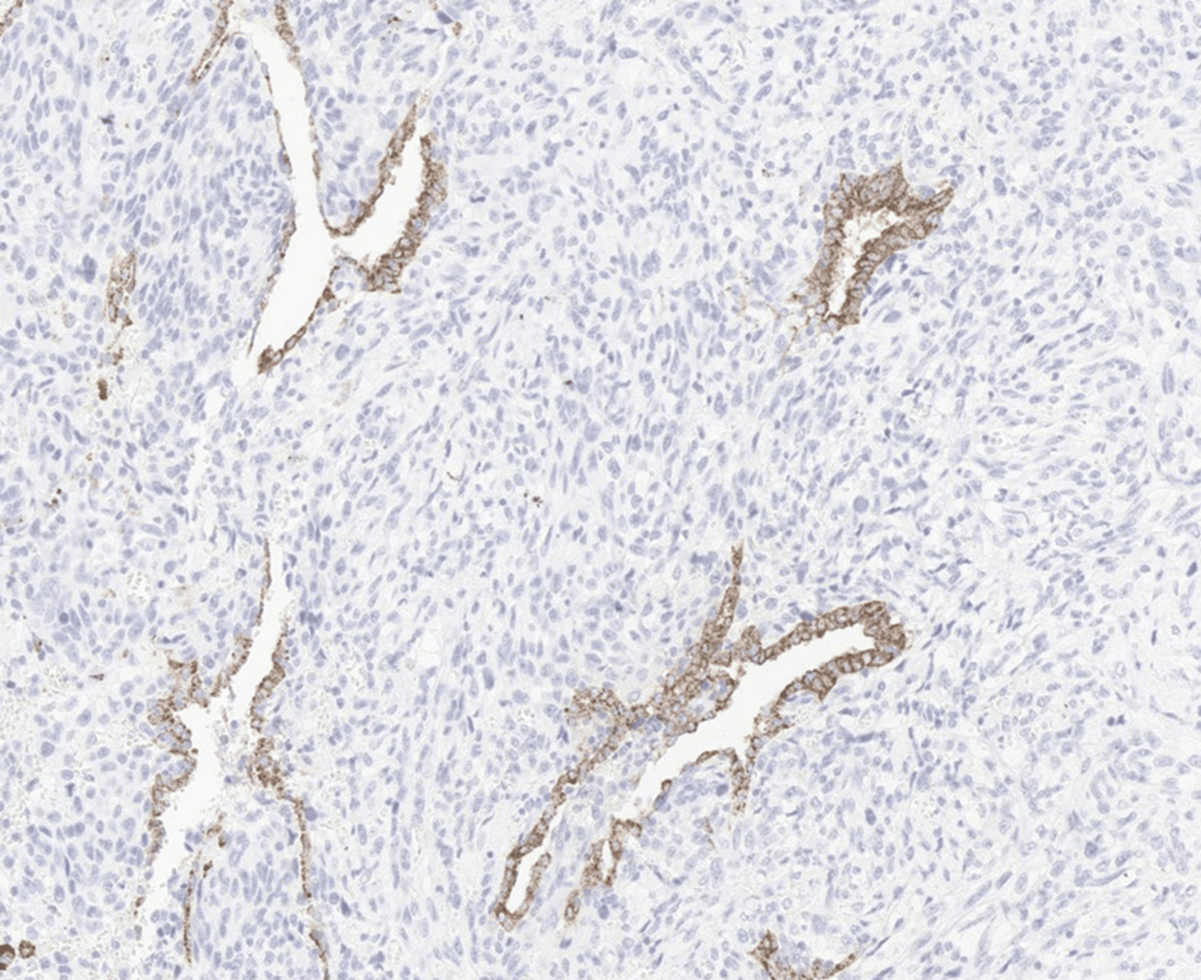
Immunohistochemical staining highlighting epithelial tumor components Immunohistochemical staining for Napsin A highlights the epithelial component of the tumor, supporting the diagnosis of pleomorphic carcinoma with mixed epithelial and sarcomatoid differentiation.

Following multidisciplinary discussion, the patient underwent robotic right lower lobectomy with systematic mediastinal lymph node dissection. Surgical pathology (Figure 5,6) revealed a 4.2-cm pleomorphic carcinoma composed predominantly of spindle cells with pleomorphic giant cells and focal epithelial components. The tumor was partially cystic and necrotic with extensive lymphovascular invasion but no visceral pleural invasion. Surgical margins were negative. Fourteen mediastinal and hilar lymph nodes were negative for carcinoma, resulting in a final pathologic stage of pT2bN0M0 (stage IIA). Background lung tissue demonstrated emphysema with foci of non-mucinous adenocarcinoma in situ and atypical adenomatous hyperplasia. Immunohistochemistry showed Napsin A positivity within epithelial tumor components (Figure 7).

Comprehensive molecular profiling identified a TP53 mutation, high tumor mutational burden, and a PD-L1 tumor proportion score of 95%, without actionable driver mutations. Given the aggressive histology and high-risk features despite node-negative disease, adjuvant cisplatin-pemetrexed chemotherapy was initiated, with plans for one year of maintenance atezolizumab immunotherapy. At approximately three months of follow-up after initiation of adjuvant therapy, the patient was tolerating treatment well without dose-limiting toxicities or recurrence of pneumothorax.

## Discussion

Pulmonary sarcomatoid carcinoma is a rare but highly aggressive form of NSCLC associated with poor prognosis and limited responsiveness to traditional chemotherapy. Pleomorphic carcinoma, the most common subtype, is characterized by histologic heterogeneity and aggressive clinical behavior. Most patients present with advanced disease, making early-stage diagnosis and curative-intent treatment uncommon [2,3].

Spontaneous pneumothorax is an uncommon initial presentation of primary lung cancer, reported in only 0.03-0.05% of cases [12]. When pneumothorax occurs in association with malignancy, proposed mechanisms include rupture of tumor-associated cavitary lesions into the pleural space, tumor necrosis with pleural disruption, bronchial obstruction causing a check-valve effect and distal airspace overdistension, or rupture of adjacent emphysematous bullae [8,12,13]. In this patient, the pneumothorax likely represented a secondary spontaneous pneumothorax occurring in a structurally abnormal lung, with possible contribution from cavitary tumor rupture given the lesion's proximity to the pleura.

Diffuse cystic lung disease significantly complicated the diagnostic process. The radiographic appearance prompted consideration of a broad differential diagnosis, including smoking-related emphysema, pulmonary Langerhans cell histiocytosis, lymphangioleiomyomatosis (LAM), and autoimmune-associated interstitial lung disease. The pathologic and biochemical findings were not in keeping with LAM or pulmonary Langerhans cell histiocytosis. Although positive SSA and SSB antibodies raised concern for Sjögren syndrome-associated lung involvement and a paraneoplastic autoimmune process, the absence of sicca symptoms, normal complement levels, and lack of lymphoid pathology on resection suggested these serologic findings were incidental. Smoking-related parenchymal injury, therefore, remains the most plausible explanation for the diffuse cystic changes.

This case emphasizes the importance of maintaining a high index of suspicion for malignancy in patients presenting with pneumothorax when imaging demonstrates atypical features such as thick-walled cavities, irregular margins, or focal lesions superimposed on diffuse lung disease. Advanced bronchoscopic techniques enabled timely tissue diagnosis and staging, facilitating curative-intent surgical management.

Surgical resection remains the cornerstone of treatment for localized PSC and is the strongest favorable prognostic factor [3,14]. Despite node-negative disease, this patient's tumor exhibited multiple high-risk features, including size greater than 4 cm, pleomorphic histology, and extensive lymphovascular invasion, supporting the use of adjuvant systemic therapy. Molecular profiling has increasingly informed treatment decisions in PSC, as these tumors frequently exhibit TP53 mutations, high tumor mutational burden, and marked PD-L1 overexpression [15]. Retrospective studies suggest that immune checkpoint inhibitors may offer improved outcomes in PSC compared with conventional chemotherapy, particularly in tumors with high PD-L1 expression [16,17].

The decision to proceed with lobectomy despite diffuse cystic lung disease highlights the importance of physiologic assessment when evaluating operability. Pulmonary function testing demonstrated preserved ventilatory capacity, underscoring that radiographic appearance alone should not preclude surgical consideration.

## Conclusions

Pulmonary sarcomatoid carcinoma may present in an atypical manner, masquerading as benign pulmonary pathology such as spontaneous pneumothorax in the setting of diffuse cystic lung disease. Early tissue diagnosis, multidisciplinary evaluation, and integration of molecular profiling are critical to achieving optimal outcomes.
